# Can normal breath sounds in mechanically ventilated patients be termed vesicular?

**DOI:** 10.1186/s13054-023-04667-4

**Published:** 2023-09-30

**Authors:** Sai Saran, Saumitra Misra, Avinash Agrawal, Suhail Sarwar Siddiqui

**Affiliations:** Department of Critical Care Medicine, King George Medical University, Lucknow, Chowk, Uttar Pradesh 226003 India

Sir,

Typically, textbooks of bedside clinical medicine state that vesicular breath sounds constitute the auscultation of the normal respiratory system. The contention of this article is that the breath sounds heard during the auscultation of mechanically ventilated patients in the intensive care unit (ICU) and operating room (OR) (without significant lung pathology on clinical examination) may not be classified as vesicular breath sounds. We argue that usual breath sounds heard upon the auscultation of the respiratory system of the mechanically ventilated patient may be more correctly termed as “usual ventilatory breath sounds or mechanical breath sounds”. To this end, we review the characteristics of vesicular breath sounds and elucidate the differences in flow patterns in mechanical ventilated patients vis-à-vis normal subjects, which may explain as to why, it is challenging to appreciate vesicular breath sounds in this special population.

As described classically, vesicular breath sounds as soft, low-pitched (< 200 Hz), with an inspiratory/expiratory ratio (I:E) of about 2:1. The expiration is short, and there is no pause between inspiration and expiration [1]. The usual flow rates at rest during tidal breathing range from 5–10 L/min, and during exercise, these may reach up to 30–40 L/min producing such a characteristic vesicular breathing pattern in healthy lungs [2]. In comparison, bronchial breath sounds are loud, harsh, and high-pitched (> 400 Hz), heard normally over the trachea. In mechanically ventilated patients with normal lungs, the minimal inspiratory flow rate range between 45 and 60 L/min (Table 1). In addition, with added leaks of up to 25% and in cases with coexisting acute respiratory distress syndromes (ARDS), very high peak inspiratory flow rates up to 200 L/min may be needed. Normal physiological flow pattern is sinusoidal but usual flow patterns in various ventilators are of descending ramp or square wave flow pattern or rarely ascending ramp, gas delivery is altered, and turbulent flow is generated, which is unlikely to result in soft and low-pitched sounds which are characteristic of vesicular breath sounds. Moreover, the I:E ratio set by default in ICU/OR ventilator is 1:2, inverse of the described I:E ratio in vesicular breath sounds. In most ventilators, inspiratory pause is set, again different from the described lack of inspiratory pause in normal vesicular breath sounds [3]. Table 1 highlights the key factors why vesicular breath sounds cannot be heard in patients on mechanical ventilation. Therefore, expecting a normal physiology in patients on invasive mechanical ventilation is further not possible as during normal quiet breathing air passes through various humidification systems through the nose and sinuses to reach the trachea, whereas the air/oxygen in ventilation is pushed through a turbine/piston/bellows to the trachea directly through a narrow tube, generating flows up to 240L/min [4]. Apart from this, performing auscultation in critically ill patients in noisy environments is challenging, with point-of-care lung ultrasound almost replacing the stethoscope, in several clinical scenarios [5].

**Table 1:** Differences between vesicular and mechanical breath sounds

S. no.	Characteristic	Vesicular breath sounds	Mechanical breath sounds
1	Intensity	Soft	Harsh
2	Flow pattern	Sinusoidal	Descending ramp, square wave
3	Pitch	Low-pitched	High-pitched
4	Inspiration-to-expiration ratio	2:1	1:2
5	Pause between inspiration and expiration	No	Usually set on a ventilator (inspiratory pause)
6	Inspiratory flow rates	20–30 L/min	45-60L/min

To conclude, we wish to reiterate that the term “vesicular” breath sounds be avoided in the context of typically heard breath sounds in the mechanically ventilated patient, and instead, this term be replaced by the more accurate terms such as “usual ventilator breath sounds or mechanical breath sounds.”

## Data Availability

Not applicable.
